# Calcium Chelation by Alginate Activates the Type III Secretion System in Mucoid *Pseudomonas aeruginosa* Biofilms

**DOI:** 10.1371/journal.pone.0046826

**Published:** 2012-10-08

**Authors:** Shawn R. Horsman, Richard A. Moore, Shawn Lewenza

**Affiliations:** Department of Microbiology and Infectious Diseases, University of Calgary, Calgary, Alberta, Canada; Charité-University Medicine Berlin, Germany

## Abstract

The extracellular biofilm matrix includes primarily DNA and exopolysaccharides (EPS), which function to maintain aggregate structures and to protect biofilms from antibiotics and the immune response. Both polymers are anionic and have cation binding activity, however the impact of this activity on biofilms is not fully understood. Host cell contact is considered the primary signal for activation of most type III secretion systems (T3SS), although calcium limitation is frequently used as a trigger of contact-independent T3SS expression. We hypothesized that alginate, which is a known calcium binding exopolysaccharide produced in mucoid *Pseudomonas aeruginosa* isolates, can activate the T3SS in biofilms. The addition of exogenous purified alginate to planktonic, non-mucoid PAO1 cultures induced expression of *exoS*, *exoT* and *exoY-lux* reporters of the T3SS in a concentration-dependent manner. Induction by alginate was comparable to induction by the calcium chelator NTA. We extended our analysis of the T3SS in flow chamber-cultivated biofilms, and showed that hyperproduction of alginate in *mucA22* mucoid isolates resulted in induction of the *exoS-gfp* transcriptional reporter compared to non-mucoid paired isolates. We confirmed the transcriptional effects of alginate on the T3SS expression using a FlAsH fluorescence method and showed high levels of the ExoT-Cys_4_ protein in mucoid biofilms. Induction of the T3SS could be prevented in planktonic cultures and mucoid biofilms treated with excess calcium, indicating that Ca^2+^ chelation by the EPS matrix caused contact-independent induction. However, mucoid isolates generally had reduced *exoS-lux* expression in comparison to paired, non-mucoid isolates when grown as planktonic cultures and agar colonies. In summary, we have shown a mucoid biofilm-specific induction of the type III secretion system and highlight a difference between planktonic and biofilm cultures in the production of virulence factors.

## Introduction

Biofilms are multicellular, surface-associated microbial communities encased in an extracellular matrix largely composed of extracellular DNA and exopolysaccharides (EPS) [1], [2]. Both DNA and EPS are structural components of the biofilm matrix that are required for attachment, aggregation and the later stage of biofilm maturation [1], [3]. In mucoid isolates of *P. aeruginosa*, alginate is hyperproduced and is involved in the formation of biofilms with highly structured architecture [4]. The *pel* and *psl* gene clusters code for the production of the primary exopolysaccharides in non-mucoid strains that are required for attachment to plastic surfaces and epithelial cells, as well as aggregation and pellicle formation [3]. Exopolysaccharides also perform capsule functions that include reducing phagocytosis by macrophages [5], [6], limiting neutrophil migration, preventing the binding of complement factors and absorbing reactive oxygen species [6]. Another property of the EPS matrix is to provide short-term protection as a diffusion barrier to antibiotics, although not all antibiotics have reduced penetration through biofilms [7].

Cation chelating activity is a common property of extracellular, anionic polymers present in the biofilm matrix. Alginate is the EPS polymer hyperproduced in mucoid Cystic Fibrosis (CF) isolates of *P. aeruginosa* and has a cation chelating activity, with a higher binding affinity for Ca^2+^ than for Mg^2+^ [8]. Purified alginate from mucoid *P. aeruginosa* was shown to bind magnesium with weak interactions and preferentially binds calcium leading to cation-induced cross-linking and gelation of alginate [8]. It has been reported that Ca^2+^ binding activity is a general property of exopolysaccharides isolated from several bacterial species [9]. We recently identified a novel function of extracellular DNA as a chelator of divalent metal cations, including Mg^2+^, Ca^2+^, Zn^2+^ and Mn^2+^ [10]. The magnesium binding activity of DNA was required for induced expression of the *arn* antimicrobial peptide resistance genes and increased antimicrobial resistance in biofilms [10].

The type III secretion system (T3SS) is a conserved virulence mechanism found in Gram-negative bacteria and is required for cytotoxicity and immune evasion [11], [12]. The T3SS uses a needle-like structure to deliver effector proteins across the two membranes of the Gram-negative envelope and directly into the cytoplasm of host cells, where they are then able to exert their toxic effect. *P. aeruginosa* encodes four effector proteins translocated by the type III secretion system: ExoS, ExoT, ExoU and ExoY [13]. Contact with the host cell is considered the most relevant cue for triggering T3SS in the context of an infection, however Ca^2+^ limitation is routinely used in vitro to trigger type III secretion in *P. aeruginosa*, without host cell contact [14], [15]. The low Ca^2+^ response leading to increased T3SS expression is also observed in pathogenic species of *Yersinia* [16] and cation levels influence expression of the T3SS in *Chlamydia trachomatis* [17] and *Salmonella* [18].

*P. aeruginosa* grows as a biofilm in the lungs of CF patients and mucoid isolates arise in the CF lung to promote long-term survival. Several previous reports have compared the expression of the T3SS in mucoid and non-mucoid isolates and concluded that the T3SS is repressed in mucoid isolates [19], [20]. These observations support the general view that long-term, chronic isolates have adapted for reduced virulence factor production in order to evade the immune response. In all of these studies, virulence factor production was assessed in planktonic cultures, although it is generally accepted that *P. aeruginosa* grows as a biofilm in the CF lung. Here we provide evidence that alginate production in mucoid isolates can induce expression and production of type III secreted toxins during growth in flow-chamber biofilms.

## Results

### Calcium chelation by alginate induces expression of the T3SS

**Figure 1:**
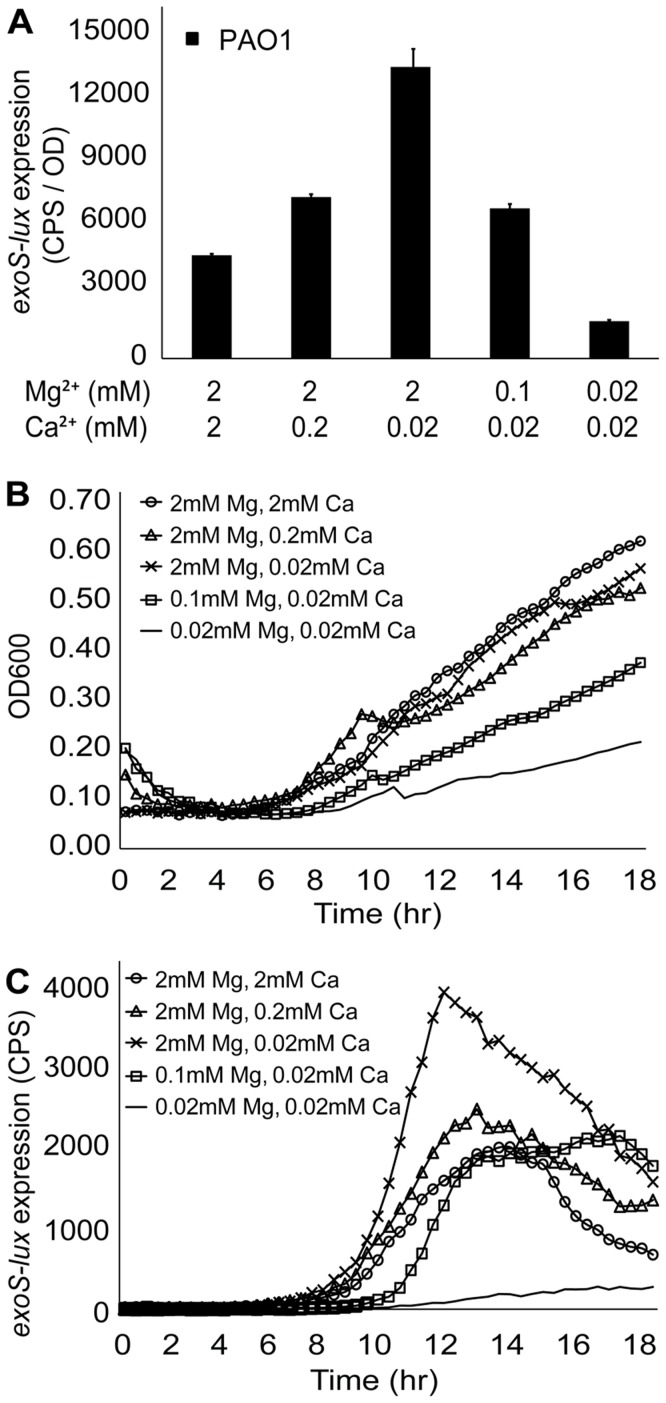
Calcium limitation induces expression of the type III secretion system in *P. aeruginosa* PAO1. (A) *P. aeruginosa* PAO1 *exoS-lux* was used as a reporter for activation of the T3SS in BM2 media supplemented with varying amounts of magnesium and calcium. Gene expression was measured every 20 minutes for 18 hours and the maximal gene expression is shown. The values shown are the means from triplicate experiments and the error bars represent the standard deviation. All values differ significantly (p<0.02 by unpaired *t*-test) from the control (2 mM Mg^2+^, 2 mM Ca^2+^). (B) Growth curves in BM2 media with corresponding magnesium and calcium concentrations. (C) Raw CPS illustrating the kinetics of *exoS-lux* expression in various magnesium and calcium concentrations. Similar curves have been observed for *exoT-lux* and *exoY-lux* expression over time (data not shown). Each experiment was performed at least three times and representative curves are shown.

We first examined expression of a transcriptional *exoS-lux* fusion in wild type PAO1 under conditions with varying cation concentrations and observed that the T3SS was maximally expressed with low levels of Ca^2+^ (20 µM) and relatively high concentration of Mg^2+^ (0.1–2 mM) (Fig. 1A,C). The T3SS was repressed with the addition of 2 mM Ca^2+^, confirming the role of calcium limitation in inducing expression of the T3SS. The T3SS was also repressed under low Ca^2+^ conditions when Mg^2+^ levels were also present in low µM levels, indicating that Mg^2+^ availability also influenced expression of the T3SS (Fig. 1A,C). There was a longer lag and a reduction in growth rate and final growth yield with µM amounts of Mg^2+^ and Ca^2+^, which may explain the repression of the T3SS under these conditions (Fig. 1B). The kinetics of gene *exoS-lux* expression over time are shown in Figure 1C and illustrate that the peak of expression occurred in mid-log phase between 11–13 hours. Similar expression kinetics were seen for *exoY-lux* and *exoT-lux* (data not shown).

**Figure 2:**
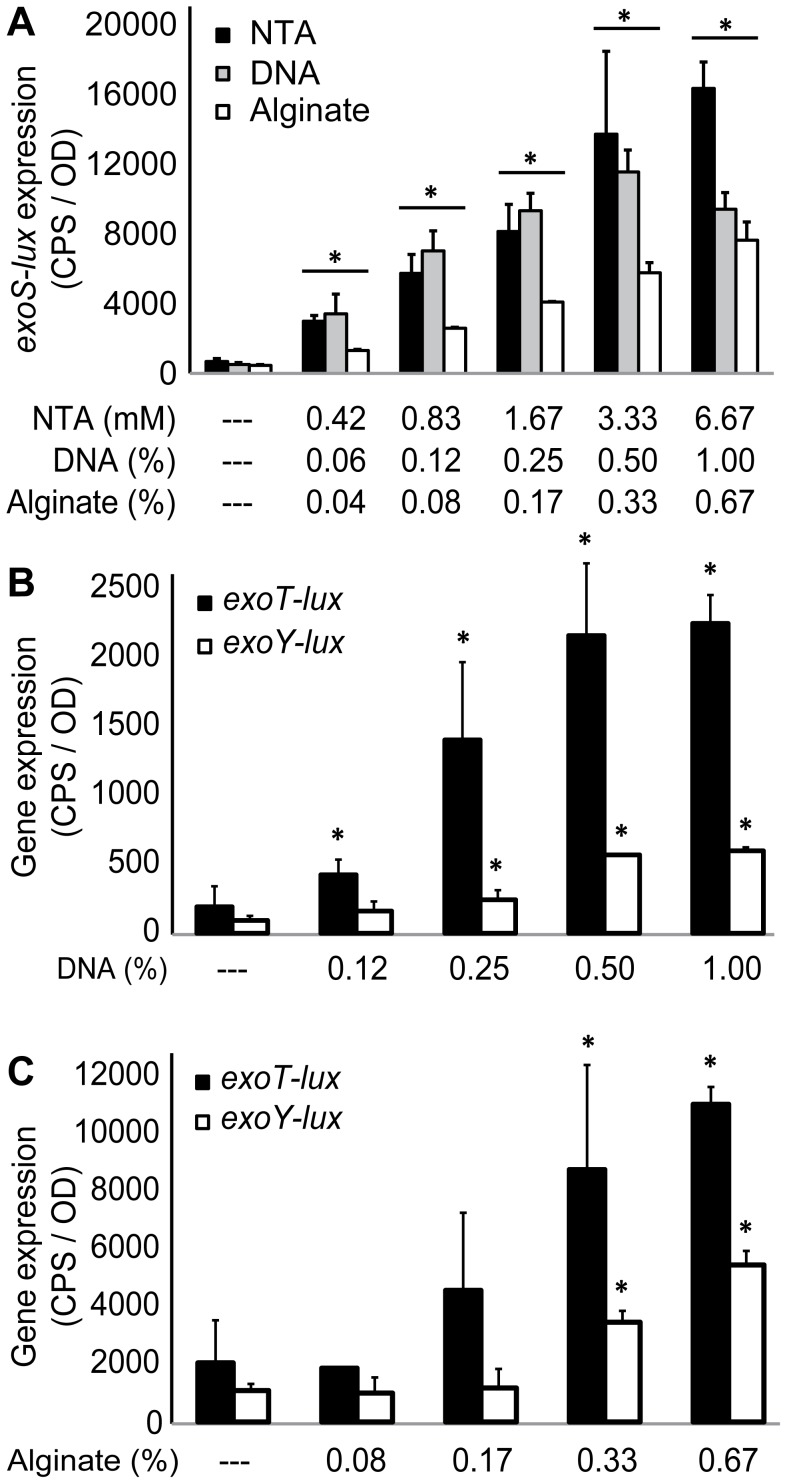
Exogenous alginate and DNA induce expression of the T3SS in *P. aeruginosa* planktonic cultures. *P. aeruginosa* PAO1 *exoS-lux*, *exoT-lux* and *exoY-lux* were used as reporter strains to monitor expression of the T3SS. (A) Bioluminescence gene expression assays were used to measure *exoS-lux* expression in planktonic cultures of PAO1 grown in BM2 with 0.2 mM Ca^2+^ and 0.5 mM Mg^2+^ and supplemented with various concentrations of NTA, exogenous DNA or alginate. (B) Gene expression assays were used to measure *exoT-lux* and *exoY-lux* expression in planktonic cultures of PAO1 grown in BM2 with 0.2 mM Ca^2+^ and 0.5 mM Mg^2+^ and supplemented with various concentrations of alginate (C) or (D) extracellular DNA. Gene expression was measured every 20 minutes for 18 hours and the maximal gene expression is shown. The values shown are the means from triplicate experiments and the error bars represent the standard deviation. Values that differ significantly (p<0.02 by unpaired *t*-test) from the controls (BM2 alone) are marked with an asterisk. Each experiment was performed at least three times and representative values are shown.

DNA and alginate have both been shown to have calcium chelating activity [8], [10], but the consequence of this property is unknown in the context of bacterial biofilms. We tested if both matrix polymers could induce expression of the type III secretion system, in a similar manner to the addition of typical Ca^2+^ chelators (EGTA, NTA) to planktonic cultures [14], [15]. Exogenous alginate was added in a range of concentrations to planktonic PAO1 cultures expressing transcriptional reporters *exoS-lux, exoY-lux and exoT-lux*. In this series of experiments, we used BM2 growth media with high Ca^2+^ (0.2 mM) and Mg^2+^ (0.5 mM), which resulted in low basal levels of *exoS*, *exoY* and *exoT*, due to the repressing levels of Ca^2+^ (0.2 mM) (Fig. 2). As a positive control, we added the known calcium chelator NTA and compared the gene expression profiles in all conditions. Exogenous alginate induced expression (up to 17-fold) of the *exoS-lux*, *exoY-lux* and *exoT-lux* reporters in a concentration-dependent manner (Fig. 2A,B). The *exoS-lux*, *exoY-lux* and *exoT-lux* reporters were also strongly induced (up to 23-fold) by exogenous DNA in a concentration-dependent manner (Fig. 2A,C). Of the three chelators, NTA and DNA caused comparable levels of induction, except at high concentrations of NTA, and alginate caused slightly lower levels of induction (Fig. 2). Expression of the T3SS reporter was induced 3-fold when calcium was decreased from 2 mM to 0.02 mM (Fig. 1A) or when alginate was added at concentrations ranging from 0.17–0.25% (Fig. 2). Higher concentrations of alginate caused up to a 17-fold induction in media with a baseline 0.2 mM calcium concentration (Fig. 2A), which illustrates the large capacity for binding and sequestering calcium.

**Figure 3:**
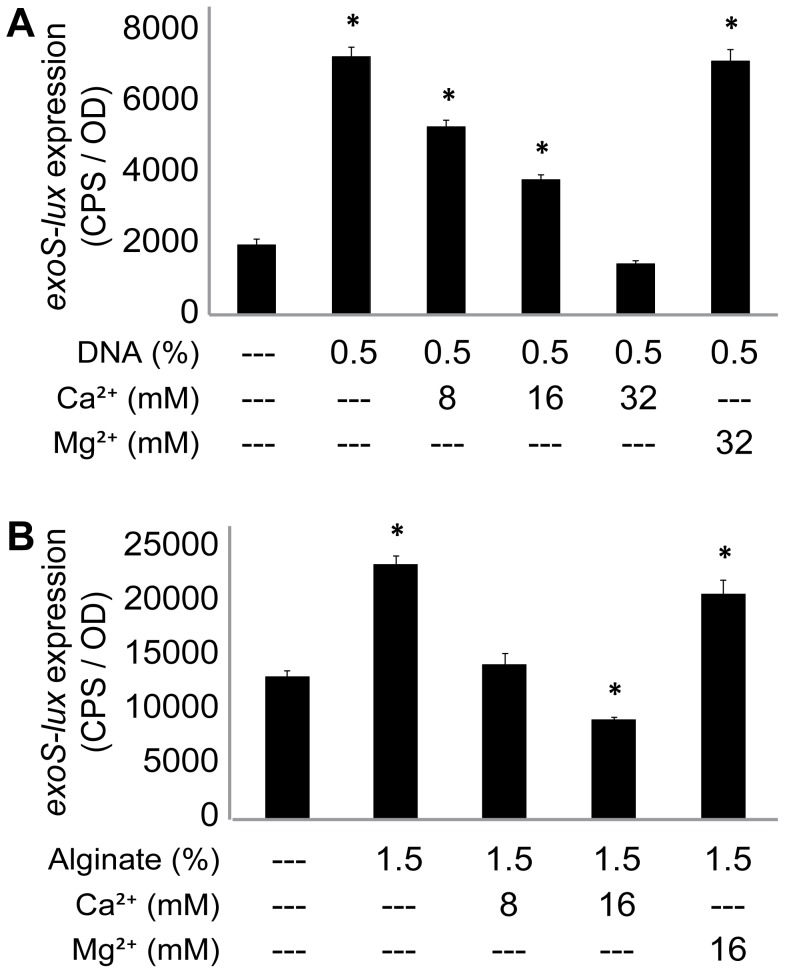
Calcium chelation by exogenous DNA and alginate induces expression of the T3SS in *P. aeruginosa*. *P. aeruginosa* PAO1 *exoS-lux* was used as a reporter for the T3SS. Planktonic cultures were supplemented with (A) exogenous DNA or (B) alginate and various concentrations of calcium and magnesium to BM2 with a baseline concentration of 0.2 mM calcium. Gene expression was measured every 20 minutes for 18 hours and the maximal gene expression is shown. The values shown are the means from experiments done in triplicate and the error bars represent the standard deviation. Values that differ significantly (p<0.02 by unpaired *t*-test) from the controls (BM2 alone) are marked with an asterisk. Each experiment was performed at least three times and representative values are shown.

To determine if induction of the T3SS was due to the calcium chelating activity of alginate or DNA, excess cations were added simultaneously with each polymer and the expression of *exoS-lux* was measured. While the addition of Mg^2+^ (32 mM) had no effect on DNA-induced T3SS expression, the addition of Ca^2+^ (8 and 16 mM) significantly reduced expression and the addition of 32 mM Ca^2+^ completely abrogated the DNA-induced expression (Fig. 3A). Similarly, the addition of Mg^2+^ (16 mM) had no effect on alginate-induced T3SS expression, but the addition of Ca^2+^ (8 and 16 mM) completely abrogated the induction by alginate (Fig. 3B). Together, these data suggest that alginate and DNA have a great capacity to sequester calcium, and that calcium chelation was specifically required for induction of the T3SS in this planktonic culture system with exogenous polymer.

### Comparison of *exoS-lux* expression in planktonic cultures and colonies in mucoid and non-mucoid strains

**Figure 4:**
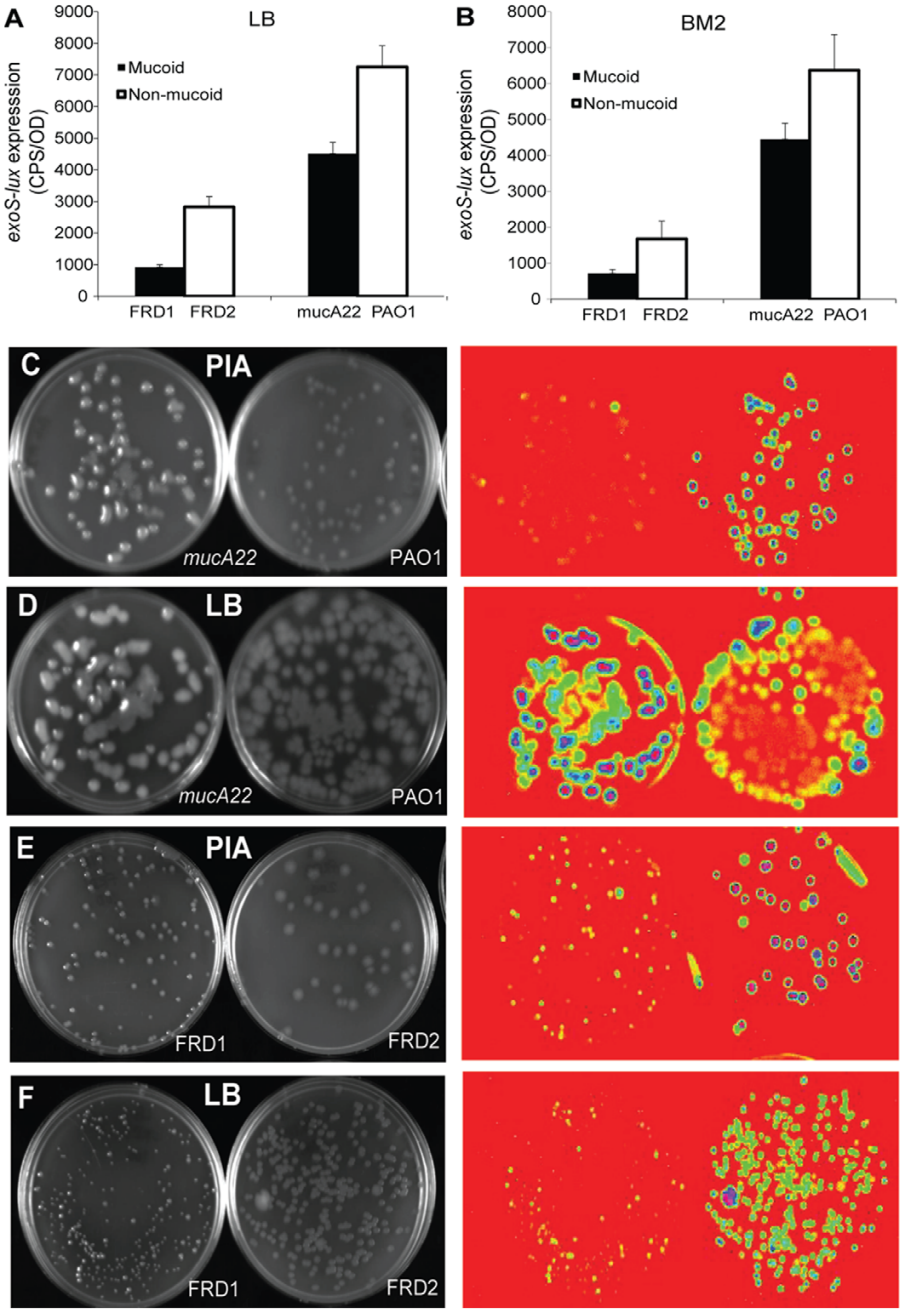
*ExoS-lux* expression in mucoid and non-mucoid isolates grown in planktonic cultures and as agar colonies. (A) Mucoid (FRD1, PAO1*mucA22*) and non-mucoid (FRD2, PAO1) *P. aeruginosa* strains encoding a chromosomal *exoS-lux* fusion as a reporter for the T3SS were grown in LB and (B) BM2 (0.5 mM Mg^2+^, 0.2 mM Ca^2+^) planktonic cultures. Gene expression was measured every 20 minutes for 18 hours and the maximal gene expression is shown. The values shown are the means from experiments done in triplicate and the error bars represent the standard deviation. All values from mucoid strains (FRD1, mucA22) differ significantly (p<0.02 by unpaired *t*-test) from the corresponding non-mucoid controls (FRD2, PAO1). (C,E) This strain panel was plated on Pseudomonas Isolation Agar (PIA) and (D,F) LB and incubated at 37°C for 2 days. Bioluminescence was detected in Bio-Rad XRS Chemidoc Imaging system. The colonies are depicted using epi white illumination (left panel) and corresponding *exoS-lux* expression using bioluminescence imaging (lower panel). Each experiment was performed at least three times and representative values and images are shown.

To determine if the effects of exogenous alginate could be observed in strains that naturally produced alginate, we compared the expression of *exoS-lux* in both planktonic cultures and agar-grown colonies in mucoid and non-mucoid isolates of *P. aeruginosa.* For these experiments we constructed a *mucA22* mutant in PAO1, which resulted in the mucoid colony phenotype. In addition, we used the original mucoid isolate FRD1 (*mucA22; single base pair* mutation) and its non-mucoid derivative FRD2 [21]. When these cultures were grown in planktonic conditions, *exoS-lux* expression was slightly higher (∼2-fold) in non-mucoid isolates PAO1 and FRD2 (Fig. 4A,B). These results are consistent with numerous reports in the literature showing that mucoid isolates have reduced expression of the T3SS when grown in liquid, planktonic cultures [19], [20].

Next, all strains were grown on agar plates and *exoS-lux* gene expression was monitored. Colonies of the non-mucoid strains PAO1 and FRD2 were generally brighter than those of the mucoid isolates FRD1 and PAO1*mucA22* on LB or PIA agar (Fig. 4C-F) with the exception of LB agar where *exoS-lux* expression was comparable and slightly brighter in the *mucA22* mucoid isolate of PAO1. This pattern of expression between mucoid and non-mucoid strains was consistent for up to 5 days, and expression levels decreased over time (data not shown).

### Mucoid isolates induce *exoS-gfp* expression in flow chamber biofilms

**Figure 5:**
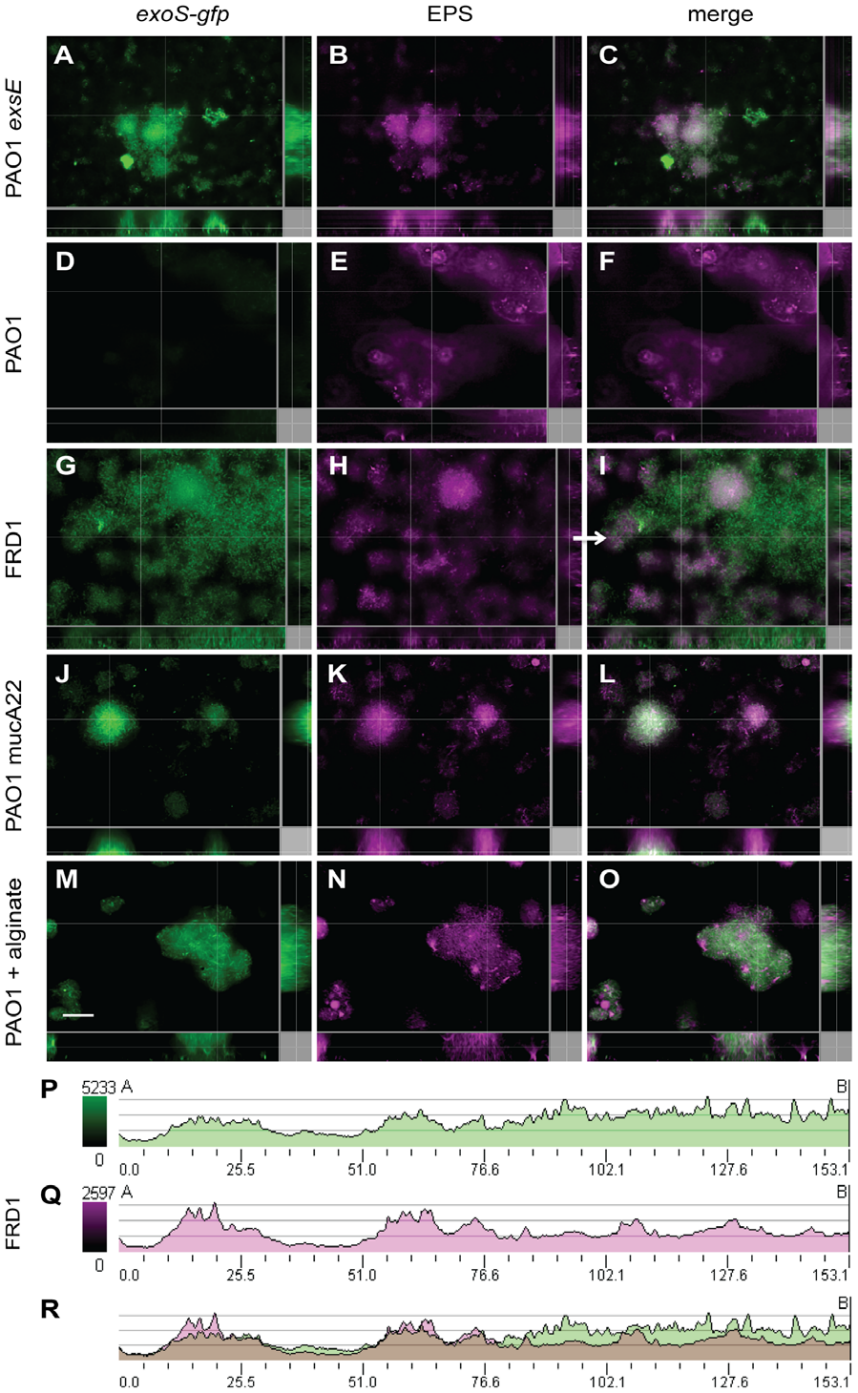
The T3SS expression is induced in flow chamber biofilms of mucoid, alginate producing *P. aeruginosa* strains. Flow chamber-grown biofilms of (A–C) constitutive T3SS expressing strain PAO1 Δ*exsE*, (D–F) non-mucoid PAO1, (G–I) mucoid FRD1, (J–L) mucoid PAO1 *mucA22* and (M–O) non-mucoid PAO1 with the exogenous addition of 10 mg/ml alginate, all encoding *exoS-gfp* were cultivated at 37°C for 2 days. Fluorescence microscopy shows the level of *exoS-gfp* expression in each strain (A, D, G, J) and the biofilm matrix counterstained with calcofluor for exopolysaccharides (B, E, H, K). The merged images are provided in the right column (C, F, I, L,O). In each case, representative images of x-y (large panel), x-z (bottom panel), and y-z (right panel) slices are displayed. Scale bar, 15 µm. The signal intensity was plotted for the green (*exoS-gfp*) (P) and purple (EPS) (Q) channels along the line indicated by the white arrow in the x-y plane displayed for FRD1 in panel I. The merged graph is provided (R), with the signal intensities overlapping for the green and purple channels to examine co-localization. Each experiment was performed at least three times and representative images are shown.

To determine the influence of the biofilm matrix polymers on expression of the T3SS in *P. aeruginosa*, we extended our analysis to flow chamber-cultivated biofilms. We constructed a T3SS reporter strain to express a chromosomally encoded *exoS-gfp* fusion to visualize expression of the T3SS in biofilms. All biofilms were cultivated in flow chamber devices for 2 days at 37°C, which generally resulted in biofilms with a 20–30 µM thickness. As a positive control, the strain PAO1Δ*exsE* that constitutively expresses the T3SS [22] was used to demonstrate high levels of *exoS-gfp* expression in biofilms (Fig. 5A–C). In contrast, *exoS-gfp* was not expressed in the non-mucoid strain PAO1 (Fig. 5D–F) or PAK (data not shown). Staining the EPS with calcofluor was sufficient to indicate biofilm structure, despite the lack of *exoS-gfp* expression in PAO1. Although extracellular DNA accumulated in the matrix of PAO1 biofilms (data not shown) and the Pel and Psl exopolysaccharides are produced in this strain [3], neither of these matrix components were sufficient to induce T3SS expression in non-mucoid PAO1 flow chamber biofilms.

Next, we introduced the *exoS-gfp* transcriptional fusion onto the chromosome of FRD1 [21] and a *mucA22* mucoid isolate derived from PAO1. In contrast to that seen in non-mucoid strains, *exoS-gfp* was highly expressed in the mucoid strains FRD1 (Fig. 5G–I) and PAO1 *mucA22* (Fig. 5J–L). To determine if exogenous alginate could induce the T3SS in non-mucoid PAO1 biofilms, we examined PAO1 biofilms cultivated in the presence of exogenous alginate for *exoS-gfp* expression. In agreement with the naturally mucoid strains, the addition of purified alginate was sufficient to induce *exoS-gfp* expression in the otherwise non-mucoid strain PAO1 (Fig. 5M–O). For the mucoid strain PAO1 *mucA22* (Fig. 5J–L) and non-mucoid PAO1 cultivated with exogenous alginate (Fig. 5M–O), there was a clear co-localization of *exoS-gfp* expression with EPS production. For mucoid strain FRD1, there were regions where *exoS-gfp* expression was more apparent than EPS production; however the signal intensities shown in Figure 5P–R indicate a co-localization of the highest signals for EPS production with high levels of gene expression.

### The T3SS effector ExoT-Cys4 is produced in mucoid biofilms

**Figure 6:**
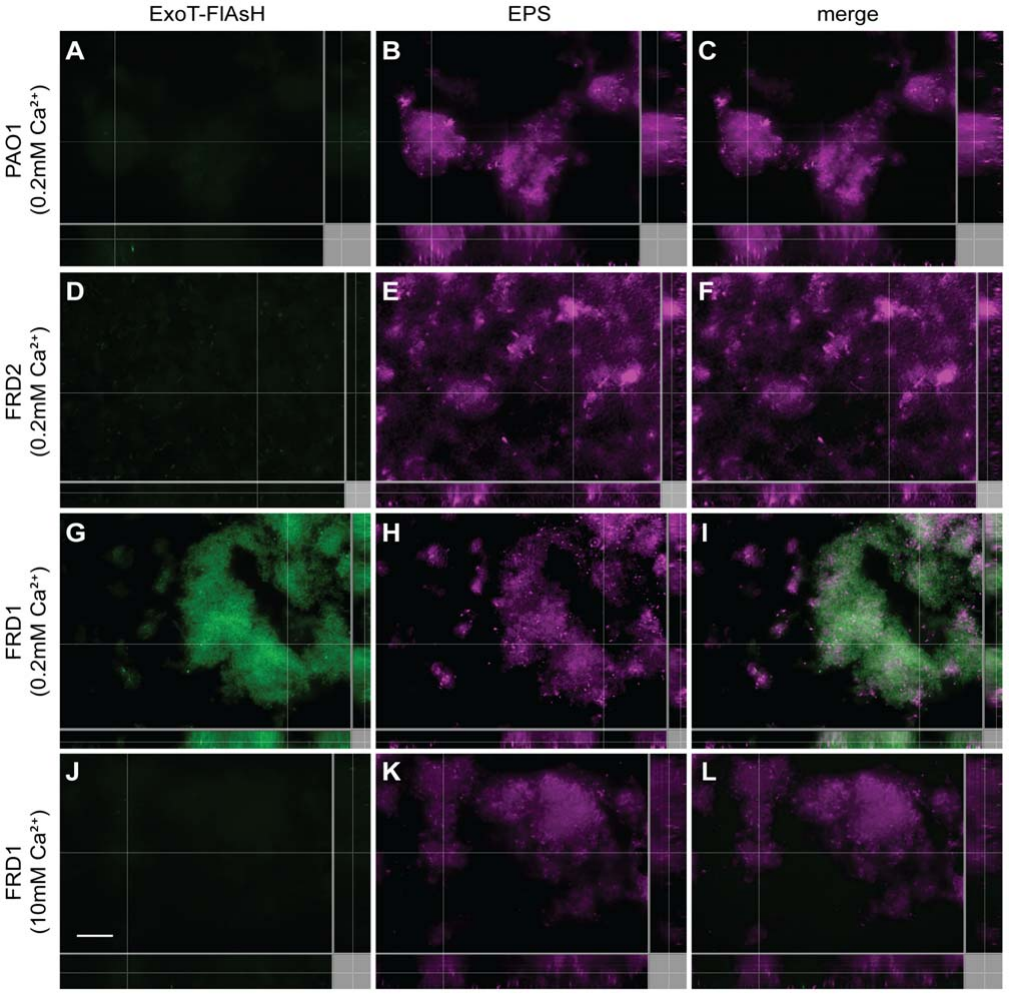
The T3SS effector protein ExoT is produced and detected in mucoid *P. aeruginosa* biofilms. ExoT was tagged with a C-terminal tetracysteine motif that can be bound by a fluorescein-based biarsenical dye (FlAsH reagent). Biofilms of non-mucoid strains PAO1 and FRD2 and mucoid strain FRD1 encoding this chromosomal ExoT*-*CCPGCC construct were cultivated at 37°C for 2 days and incubated with the FlAsH reagent. Fluorescence microscopy shows the levels of ExoT production in (A) PAO1, (D) FRD2, (G) FRD1 and (J) FRD1 grown in the presence of 10 mM exogenous calcium as well as the biofilm matrix counterstained for exopolysaccharides (B, E, H, K). The merged images are provided in the right column (C, F, I, L). In each case, representative images of x-y (large panel), x-z (bottom panel), and y-z (right panel) slices are displayed. Scale bar, 15 µm. Mucoid FRD1 biofilms were cultivated then supplemented with excess 10 mM Ca^2+^ (J–L) to examine the effect on ExoT production. Each experiment was performed at least three times and representative images are shown.

We wanted to verify that transcription of the T3SS led to production and secretion of T3SS effectors into the biofilm using fluorescence microscopy. Since translational fusions of GFP to the T3SS effectors are not secreted through the needle [23], we used a novel method to visualize the accumulation of type III effectors in *P. aeruginosa* biofilms. For this experiment, we examined the production and accumulation of ExoT in biofilms. We constructed a C-terminal tetra-cysteine tagged ExoT-Cys_4_ and expressed this protein from the chromosome. Biofilms were cultivated for 2 days at 37°C and visualized after injection of the FlAsH reagent. FlAsH is a fluorescein–based dye that binds specifically to CCXXCC amino acid sequences, a motif of four cysteines [24]–[26]. Using FlAsH microscopy, we observed that there was very little FlAsH staining of non-mucoid PAO1 (Fig. 6A–C) and FRD2 (Fig. 6D–F) biofilms expressing ExoT-Cys_4_. This result was consistent with a lack of *exoS-gfp* gene expression in non-mucoid strains (Fig. 5D–F). Likewise, there was very little staining of cells in control FRD1 biofilms not expressing the ExoT-Cys_4_ protein (data not shown), revealing limited background fluorescence in the mucoid strain FRD1 from non-specific FlAsH binding.

In contrast to the non-mucoid PAO1 (Fig. 6A–C) and FRD2 biofilms (Fig. 6D–F), significant levels of ExoT-Cys_4_ protein were detected in biofilms of mucoid strain FRD1, which hyperproduces alginate [21] (Fig. 6G–I), with a clear co-localization of ExoT-Cys_4_ detection with EPS production (data not shown). Since it is known that induced expression of the T3SS is coupled with active secretion in *P. aeruginosa* [13], [27], we propose that detection of T3SS effector ExoT indicates the secretion and accumulation of protein in the matrix of mucoid biofilms.

Increased expression and secretion of the T3SS effectors correlated with the production of alginate and we propose that activation of the T3SS is a consequence of the Ca^2+^ chelating activity of alginate. To test this hypothesis, mucoid biofilms were cultivated for 7 hours and then 10 mM calcium was added into the media and pumped across the biofilms for the remaining 35 hours, which had no effect on biofilm depth. The addition of 10 mM calcium to pre-formed biofilms was sufficient to repress expression of the T3SS in the mucoid FRD1 isolate (Fig. 6J–L).

**Figure 7:**
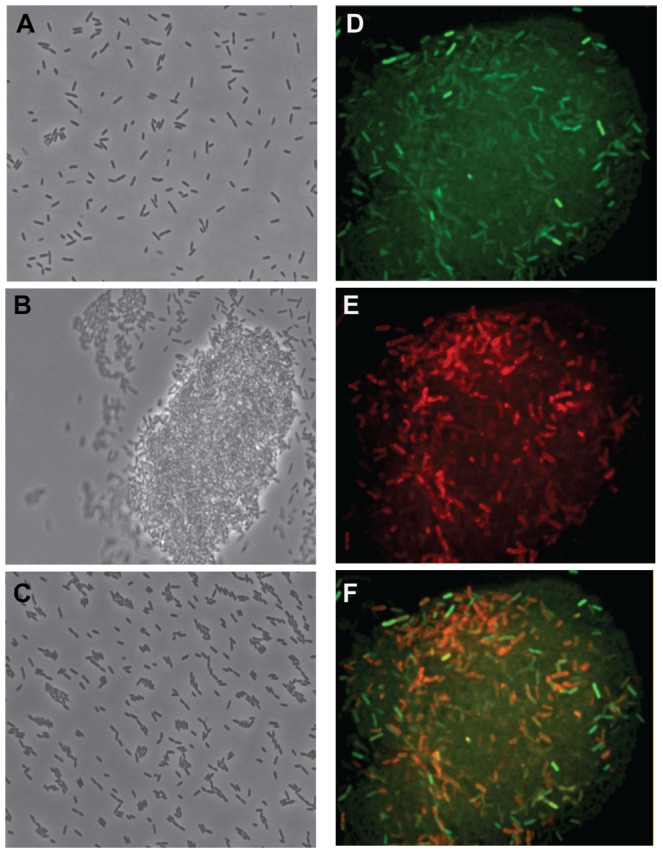
FlAsH detection of ExoT-Cys_4_ in an aggregate of the mucoid isolate FRD1. Phase contrast images of (A) PAO1, (B) FRD1 and (C) FRD2 mid-log planktonic cultures. Fluorescence microscopy shows a mid-log culture of mucoid FRD1 stained with (D) FlAsH to detect the production of ExoT-Cys_4_ and (E) a membrane-specific dye FM 4–64 (red). The merged image is shown in (F). Each experiment was performed at least three times and representative images are shown.

To examine the pattern of FlAsH staining of ExoT-Cys_4_ in greater detail, we used the FlAsH reagent in combination with a membrane-specific stain. Mid-log cultures of mucoid FRD1 were shown to produce large aggregates (Fig. 7B), whereas non-mucoid PAO1 (Fig. 7A) and FRD2 (Fig. 7C) did not aggregate in planktonic cultures. Aggregates of the mucoid strain FRD1 were stained with the membrane-specific lipophilic dye FM4-64 and the FlAsH reagent was added to detect ExoT-Cys_4_. Green FlAsH fluorescence was observed within certain individual cells and outside of individual cells, which resulted in a diffuse ExoT-Cys_4_ staining pattern in the aggregate (Fig. 7D–F). This dual staining pattern for ExoT-Cys_4_ by the FlAsh reagent indicates that extracellular and intracellular protein can be detected under these labeling conditions.

## Discussion

Alginate is an anionic polymer consisting of blocks of 1–4 linked D-mannuronate and L-guluronate residues. The negatively charged carboxylate groups are possible sites of calcium binding, which may be a general feature of bacterial exopolysaccharides [9]. Alginate is hyperproduced in mucoid CF isolates and accumulates during biofilm formation. Here we assessed the influence of alginate accumulation on expression of the type III secretion system in planktonic, agar-grown colonies and flow chamber biofilms.

In our initial experiments, we added exogenous purified alginate to planktonic cultures and monitored the effect on expression of the *exoS*, *exoY* and *exoT* type III secreted effectors. Alginate acted as a calcium chelator leading to the induction of all three effectors in a concentration-dependent manner. We then examined the influence of natively produced alginate on expression of the T3SS. Using multiple paired mucoid and non-mucoid isolates, we showed that expression of the type III secretion was slightly reduced in mucoid isolates grown as both planktonic cultures and agar colonies, compared to non-mucoid isolates. This result contrasted our experiments with exogenous polymer, but was consistent with many previous reports comparing the mucoid and non-mucoid isolates [19], [20].

Very few studies examine the production of virulence factors during biofilm growth. Therefore, we extended our analysis of the T3SS to flow chamber-cultivated biofilms. We used multiple live-imaging microscopy techniques to demonstrate that the T3SS is indeed highly expressed specifically in mucoid biofilms (Fig. 5), which results in high levels of the ExoT-Cys_4_ type III secreted-effector in mucoid biofilms (Fig. 6). While both exogenous polymers DNA and alginate induced expression of *exoS-lux* when added to planktonic cultures (Fig. 1), only alginate production was required for induction of the T3SS in biofilms.

Calcium chelators (EGTA, NTA) are frequently used to induce the T3SS in experiments without host cells. The T3SS was repressed in high Ca^2+^ (0.2 mM), but was induced when Ca^2+^ was provided in µM concentrations and with the addition of alginate to media containing repressing levels of Ca^2+^_._ Induction of the T3SS by alginate was blocked with the addition of excess calcium but not magnesium, indicating that the calcium chelating activity of alginate is required to induce the T3SS. Similarly, the addition of excess calcium to mucoid biofilms in flow chambers also prevented expression of the T3SS. These observations suggest that alginate in the biofilm matrix binds and sequester Ca^2+^, which triggers type III secretion in the absence of host cell contact. It has also been shown previously that T3SS is induced in biofilms, leading to the production of T3SS effector proteins in the biofilm effluent [28], [29].

In contrast to the expression pattern in mucoid biofilms, planktonic cultures and agar colonies had slightly reduced expression of the T3SS in mucoid isolates under most conditions. This was consistent with multiple studies that also reported reduced gene expression of T3SS effectors in other *mucA22* mucoid *P. aeruginosa* isolates. Thus, we propose that something unique about growth in a mucoid biofilm is required for induction of the T3SS. It may be related to increased EPS production or accumulation of alginate in microcolonies under flow growth conditions, or to the unique structure and organization of mucoid biofilms compared to mucoid isolates grown in liquid or on agar. Alternatively, mucoid cultures aggregate in planktonic conditions (Fig. 7), which may impose nutrient limitation or stress in planktonic cultures. It is known that metabolic stress resulting in an increased demand of certain metabolites represses the T3SS [13]. In contrast, flow chamber biofilms may not experience this stress as the biofilms are exposed to a constant flow of fresh growth media. Since it is widely accepted that *P. aeruginosa* grows as a biofilm in the CF lung [30], we propose that studying the expression of virulence genes in cultivated biofilms will yield a new understanding of the virulence traits expressed during chronic CF infections.

The detection of T3SS effector proteins in biofilms suggests that T3SS effectors may function as extracellular toxins, in addition to their functions as injected, intracellular toxins. There are several reports to support the claim that T3SS effectors have biological activity as extracellular toxins. Purified ExoS was added exogenously to immune cells and shown to stimulate monocytes to produce proinflammatory cytokines and chemokines [31] and activate T cells, resulting in proliferation and apoptosis [32]. There is mounting evidence in other type III secretion systems that extracellular effectors play a role in bacterial pathogenesis. The T3SS effector YopM from *Yersinia* was purified and shown to promote its own entry into multiple cell types and internalization of YopM resulted in reduced proinflammatory cytokine production [33]. Multiple Yop T3SS effectors of *Y. pseudotuberculosis* were found on the bacterial surface and it was proposed that translocation of Yop effectors via a T3SS-dependent delivery involves a second step to translocate ‘presecreted’ surface effectors into the host [34]. Taken together, the type III secretion system may not be exclusively required for injection of secreted effectors into host cells.

The biofilm matrix polymers have well-established functions in supporting biofilm structure and immune evasion. Here we show that the calcium binding activity of alginate can induce virulence gene expression. We have previously shown that the magnesium chelating activity of DNA can induce the expression of antimicrobial peptide resistance genes [10]. The expression of these matrix-induced phenotypes may contribute to the long-term survival of biofilms from antibiotic treatment and the immune system, and likely the ability to cause disease in chronic infections.

## Materials and Methods

### Bacterial strains and media

Strains, plasmids and primers used in the study are listed in Table 1. Unless otherwise indicated, biofilms and planktonic cultures were grown in Basal Minimal Medium (BM2) at 37°C. BM2 contained the following components: 100 mM HEPES, 7 mM (NH_4_)_2_SO_4_, 1.03 mM K_2_HPO_4_, 0.57 mM KH_2_PO_4_, 500 µM MgSO_4_, 200 µM CaCl_2_, 10 µM FeSO_4_ and ion solution containing 1.6 µM MnSO_4_·H_2_0, 13.9 µM ZnCl_2_, 4.7 µM H_3_BO_3_ and 0.7 µM CoCl_2_·6H_2_0.The ion solution and iron sulphate solutions were added to the media after it was autoclaved. The media was supplemented with 20 mM sodium succinate as a carbon source for all bioluminescence assays or 0.4 mM glucose for biofilm cultivation because glucose promotes large aggregate biofilms [42].

**Table 1:** Bacterial strains, plasmids and PCR primers used in the study.

Strain	Description	Reference
PAO1	Wild type *P. aeruginosa*	[35]
PAO1 *mucA22*	Mucoid *mucA22* derivative of PAO1	This study
FRD1	Mucoid CF isolate, *mucA* mutant	[21]
FRD2	Non-mucoid *algT18* revertant of FRD1	[21]
PAO1 *exoS-lux*	*exoS-lux* integrated in att site of PAO1	[36]
PAO1 *exoT-lux*	*exoT-lux* integrated in att site of PAO1	[36]
PAO1 *exoY-lux*	*exoY-lux* integrated in att site of PAO1	[36]
PAO1F *exoS-gfp*	Δ*exoS*::[*gfp lacZ*]	[37]
PAO1F Δ*exsE exoS-gfp*	Δ*exsE* Δ*exoS*::[*gfp lacZ*]	[22]
PAO1 *mucA22 exoS-gfp*	*exoS-gfp* integrated in att site of PAO1 *mucA22*	This study
FRD1 *exoS-lux*	*exoS-lux* integrated in att site of FRD1	This study
FRD2 *exoS-lux*	*exoS-lux* integrated in att site of FRD2	This study
FRD1 *exoS-gfp*	*exoS-gfp* integrated in att site of FRD1	This study
PAO1 ExoT-Cys_4_	ExoT-Cys_4_ integrated in attTn7 site of PAO1	This study
FRD1 ExoT-Cys_4_	ExoT-Cys_4_ integrated in attTn7 site of FRD1	This study
FRD2 ExoT-Cys_4_	ExoT-Cys_4_ integrated in attTn7 site of FRD2	This study

Plasmid	Description	Reference
pMS402	KmR, TpR; cloning vector	[38]
pCS01	AmpR; source of *gfp*mut3	[39]
pCDS128	TcR; mini-CTX integration vector for *exoS-lux*	[36]
mini-CTX-*lux*	TcR; mini-CTX integration vector	[40]
pTNS2	AmpR; helper plasmid	[41]
pUC18T-mini-Tn7T-Gm	AmpR, GmR; mini-Tn7T integration vector	[41]
pDONRX*mucA22*	Allelic exchange construct for *mucA22* gene	[19], [20]
mini-Tn7-ExoT-Cys_4_	Mini-Tn7 integration vector for ExoT-Cys_4_	This study
mini-CTX-*exoSgfp*	Mini-CTX integration vector for *exoSgfp*	This study

Primer	Sequence	
BamHI_gfpF	5′-AAAGGATCCATGCGTAAAGGAGAAGAACTTTTC-3′	
NotI_gfpR	5′-GTTGCGGCCGCCCAGTCTTTCGACTGAGCC-3′	
XhoI_exoSF	5′-GACTCGAGTGAACAGGTTCTGCTCGTT-3′	
BamHI_exoSR	5′-GAGGATCCGGACTCTGCTGAAGCGATTG-3′	
PstI_exoTF	5′-GCCTGCAGGGAAAGCGTCATGCTCGATTC-3′	
HindIII_exoTR	5′-GCAAGCTTGGCCAGGTCGAGGCCGCGCATTCTC-3′	
HK_Cys_4_F	5′-AGCTTGCGGGCAGCTTTCTGAACTGCTGCCCGGG CTGCTGCATGGAACCGGGCGGCCGTTAAGGTAC-3	
HK_Cys_4_R	5′- CTTAACGGCCGCCCGGTTCCATGCAGCAGCCCG GGCAGCAGTTCAGAAAGCTGCCCGCA-3′	

### Real-time gene expression in planktonic cultures

Bioluminescence assays were performed as previously described but with minor modifications [10]. Overnight cultures were grown in LB medium, washed in BM2 medium, diluted 1/1000 into 150 µl culture medium in a 96-well black plate with a transparent bottom (9520 Costar; Corning Inc.) and overlaid with 50 µl of mineral oil to prevent evaporation. Gene expression (luminescence) and growth (optical density) were measured at 37°C every 20 minutes for 18 hours by the Wallac Victor^2^ luminescence plate reader (Perkin-Elmer) and the maximal expression was retained. Biofilm matrix components, alginate (sodium salt, Sigma) or DNA (sodium salt, USB) were added exogenously to liquid media in the concentrations indicated. To degrade the EPS matrix in *retS* planktonic cultures, cellulase (from *Aspergillus niger*, Sigma) was added in the concentrations indicated.

### Biofilm cultivation in flow chambers

Biofilms were cultivated for 48 hours at 37°C in flow chambers with channel dimensions of 1×4×40 mm as previously described but with minor modifications [43]. Silicone tubing (VWR, .062 ID×.125 OD×.032 wall) was autoclaved and the system was assembled and sterilized by pumping a 0.5% hypochlorite solution through the system at 6 rpm for 1 hour using a Watson Marlow 205S peristaltic pump. The system was then rinsed at 6 rpm with sterile water and medium for 30 minutes each. Flow chambers were inoculated by injecting 400 µl of mid-log culture diluted to an OD600 of 0.02 with a syringe. After inoculation, chambers were left without flow for two hours after which medium was pumped though the system at a constant rate of 0.75 rpm (3.6 ml/hour).

### Construction of *P. aeruginosa* strains containing gfp transcriptional reporters

The *gfpmut3* gene was amplified by PCR from pCS01 using the primer pair BamHI_gfpF/NotI_gfpR, digested with BamHI/NotI and cloned into BamHI/NotI-digested pMS402 [38]. The promoter of *exoS* was amplified from genomic DNA of *P. aeruginosa* PAO1 using the primer pair XhoI_exoSF/BamHI_exoSR, digested with XhoI/BamHI and cloned upstream of *gfpmut3* in XhoI/BamHI-digested pMS402gfp. The suicide vector used for chromosome integration was constructed by cloning a PacI fragment containing the *exoS-gfp* construct into a 6 kb PacI fragment from the mini-CTX vector. The mini-CTX-*exoSgfp* construct was moved into *E. coli* SM10 and inserted into the chromosomes of *P. aeruginosa* at the *attB* site via biparental mating as previously described [44]. We constructed a mucoid *mucA22* mutant in the PAO1 background using the pDONRX*mucA22* allelic exchange construct, as previously described [19], [20].

### Construction of *P. aeruginosa* strains encoding ExoT-Cys4 for FlAsH microscopy

The *exoT* gene lacking a stop site was amplified by PCR from genomic DNA of *P. aeruginosa* PAO1 using the primer pair PstI_exoTF/HindIII_exoTR and digested with PstI/HindIII. An optimized, tetracysteine tag (AGSFLN**CCPGCC**MEPGGR) [45] with HindIII/KpnI sticky ends was generated by annealing the oligonucleotides HK_Cys_4_F and HK_Cys_4_R by incubating in a high salt solution at 95°C for 5 minutes, 70°C for 10 minutes and slowly cooling to 20°C. This tetracysteine tag was ligated to the C-terminus of *exoT* and then the ExoT-Cys_4_ construct was inserted into the KpnI/PstI-digested pUC18T-mini-Tn7T-Gm vector by ligation [41]. The mini-Tn7-ExoT-Cys_4_ construct was confirmed by PCR amplification and sequencing, and then moved into the chromosome of *P. aeruginosa* at the attTn7 site via electroporation with the helper plasmid pTNS2 [41].

### Fluorescent labeling of effector protein ExoT-Cys4 in a biofilm

Biofilms of *Pseudomonas aeruginosa* strains expressing the ExoT-Cys_4_ protein from the chromosome were cultivated as described above. A 400 µl solution of 5 µM FlAsH compound and 10 µM TCEP (reducing agent) was injected into each flow chamber and incubated for 90 minutes before washing once with a 400 µl solution of 250 µM BAL wash buffer and 40 µg/ml calcofluor in a 0.9% NaCl solution. The chambers were removed from the system and imaged using fluorescence microscopy.

### Biofilm labeling and microscopy

After biofilm cultivation, the fluorescent dye calcofluor was diluted and 400 µl was injected into the flow chambers. The chambers were removed from the system and imaged using fluorescent microscopy. Calcofluor (Sigma) was used at 40 µg/ml to counterstain the biofilm matrix, binding the β-1,3 and β-1,4 linkages in exopolysaccharides, Sytox red (Invitrogen) was used at 20 nM to counterstain the extracellular DNA in biofilms and FlAsH reagent (Invitrogen) was used at a concentration of 5 µM to stain the tetracysteine-tagged effector protein ExoT. All microscopy was done with a Leica DMI 4000 B widefield fluorescence microscope equipped with filter sets for monitoring of blue (Ex 390/40, Em 455/50), green (Ex 490/20, Em 525/36), red (Ex 555/25, Em 605/52) and far red (Ex 645/30, Em 705/72) fluorescence, using the Quorum Angstrom Optigrid (MetaMorph) acquisition software. Images were obtained with a 63×1.4 objective. Deconvolution was done with Huygens Essential (Scientific Volume Imaging B.V.) and 3D reconstructions were generated using the Imaris software package (Bitplane AG).
